# Group Antenatal Care Model as an innovative and sustainable maternal and child health service delivery in a Northern Nigerian State

**DOI:** 10.1371/journal.pgph.0005808

**Published:** 2026-02-11

**Authors:** Pius I. Christopher-Izere, Abiola Ajibola, Abimbola Phillips, Dorcas Magbadelo, Ibidun Jolaoso, Obioma Azurunwa, Francis Ogirima, Collins Imarhiagbe, Anthony Onwuegbusi, David Udanwojo, Emmanuel Udeh, Victor Obagunlu, Adebola Adekogbe, Sunday Joseph, Hamza Abubakar, Neyu Iiyasu, Adetosoye Adebanjo, Balarabe Gaya, Olayiwola Jaiyeola, Damilola Olaniyan, Lilian Anomnachi, Bankole Olatosi, Deborah Billings, Oluwafemi Adeagbo, Bolanle Oyeledun

**Affiliations:** 1 Clinical Service Unit, Centre for Integrated Health Programs, Federal Capital Territory, Abuja, Nigeria; 2 Strategic Information Unit, Centre for Integrated Health Programs, Federal Capital Territory, Abuja, Nigeria; 3 Department of Health Planning, Research & Statistics, Kaduna State Ministry of Health, Kaduna, Kaduna State, Nigeria; 4 Department of Maternal and Child Health, Kaduna State Primary Health Care Board, Kaduna, Kaduna State, Nigeria; 5 Technical Department, Technical Advice Connect, Federal Capital Territory, Abuja, Nigeria; 6 Department of Health Services Policy and Management, Arnold School of Public Health, Big Data Health Science Center, University of South Carolina, Columbia, South Carolina, United States of America; 7 Department of Health Promotion, Education and Behavior, Arnold School of Public Health, University of South Carolina, Columbia, South Carolina, United States of America; 8 Department of Community and Behavioral Health, College of Public Health, University of Iowa, Lowa City, Lowa, United States of America; 9 Office of the Chief Executive Officer, Centre for Integrated Health Programs, Federal Capital Territory, Abuja, Nigeria; PLOS: Public Library of Science, UNITED STATES OF AMERICA

## Abstract

The maternal and child health situation in developing countries remains critical and deeply concerning, These regions account for 99% of global maternal and under-five deaths. In Nigeria, uptake of critical life-saving maternal, neonatal and child health (MNCH) services, delivered through antenatal care (ANC), are sub-optimal, leading to poor outcomes. Group antenatal care (G-ANC) is an evolving antenatal care delivery model in low and medium-income countries. G-ANC models can improve quality and uptake of maternal health services.G-ANC model was implemented across 255 primary health facilities (PHFs) to ensure pregnant women receive components of a well-defined package of MNCH services at regular intervals through facilitated learning approaches delivered by trained health care workers. PHFs were selected based on predetermined criteria including availability of four health care workers, 24 hours operation, capacity to conduct antenatal and delivery services, and availability of space to conduct group activities. A total of 765 health care workers were trained and facilitated 26,769 G-ANC sessions between January 2021-February 2022 for 309,751 pregnant women who together formed a total of 23,220 cohorts. A total of 78,015 (25%) of pregnant women enrolled for the G-ANC were referred by Community Influencers. Post-intervention, at end-line there was more than two-fold increase (228%) in uptake of four-doses of Intermittent Preventive Treatment (IPT) of malaria (6.5% vs 21.5%), 152% increase in acceptance of post-partum contraceptives (11.4% vs 28.9%), and 66.4% increase in 8^th^ ANC visit (10.2% vs 16.9%). G-ANC provides opportunities to layer other high impact interventions across the RMNCAH+N continuum of care and should be scaled-up to all health facilities to improve uptake of RMNCAH+N services. Intersectoral collaboration, including relevant government agencies and other key players in the RMNCH space should seek to empower and educate communities on G-ANC to further increase ANC uptake by pregnant women.

## Introduction

Globally, over 40 percent of maternal and neonatal deaths occur in the first 24 hours after birth [1]. In Sub-Saharan Africa (SSA), maternal and under-five mortality are more than twice the global figures with maternal mortality rate (MMR) at 555 deaths/100,000 live births and under-five mortality rate (U5MR) at 76 deaths/1,000 live births [2]. According to the World Health Organization (WHO), globally, approximately 800 women die from preventable causes related to pregnancy and childbirth daily [3]. Studies have also shown that 95% of all maternal deaths occur in low and lower middle-income countries (LMICs) with 70 percent of global maternal deaths occurring in Sub Saharan Africa [4]. One in ten maternal deaths are due to malaria in endemic countries, including Nigeria, with malaria also associated with a 3–4 times increased risk of miscarriage and a substantially increased risk of stillbirth [5].

Nigeria, with a population of about 200 million people in 2018 [6] and about 7.5 million live births per year [7], has a total fertility rate of 4.8 children per woman as reported in 2023 [8]. During the same period, only 15% of married women aged 15–49 were using modern contraceptive method with variation in regional uptake of 9.3% in the North West, 12.9% in the North East, and 15% in the North Central Zone. The proportion of live births attended by skilled health personnel showed a slight increase from 45% in 2018 to 46% in the 2023–24 survey [9].

Nigeria contributes 10% of the global burden of maternal deaths with MMR of 512 deaths per 100,000 live births [10]. Nigeria’s target under Sustainable Development Goal (SDG) 3.1 is to reduce the high MMR to fewer than 70 per 100,000 live births by 2030. Similarly, the national goal for antenatal care (ANC) aligns with the World Health Organization’s recommendation of eight ANC contacts per pregnancy. For malaria prevention in pregnancy, Nigeria aims for 80% coverage of three or more doses of intermittent preventive treatment (IPTp3+).

Nigeria embarked on several initiatives aimed at improving maternal and reproductive health outcomes, including the presidential declaration of a state of emergency on maternal mortality in 2005 [11], the Save One Million lives initiative in 2012 [12] and the 2017 inauguration of a Task Force to accelerate reduction of maternal mortality in Nigeria [13]. However, this has not reflected significantly in maternal and child health outcomes. The 2018 Nigeria Demographic Health Survey [10] report showed a neonatal mortality rate (NMR) of 38 deaths per 1,000 live births, infant mortality rate of 67 deaths per 1000 live births and Under 5 Mortality Rate (U5MR) of 132 per 1,000 live births over a five-year period [14].

Antenatal care (ANC) – the entry point to maternal health – is critical in identifying women at risk of developing pregnancy related complications. ANC refers to the care provided by skilled health-care professionals to pregnant women to ensure the best health outcomes for both mother and baby during pregnancy [15]. In Nigeria, the uptake of critical life-saving maternal, neonatal and child health (MNCH) services, usually delivered through ANC is sub-optimal and lacks the desired impact in improving maternal and child health [16]. With only one-third of pregnant women attending four or more ANC visits [11], there is low uptake of intermittent Preventive Treatment in pregnancy (IPTp) – only 16.6% received three or more doses of sulfadoxine-pyrimethamine (SP), low skilled birth attendance (43.4%.) and low facility delivery (39.4%) [32].

Northern Nigeria has the lowest ANC utilization in the country [17]. In Kaduna, despite progress made in improving the 4^th^ ANC attendance from 54.1% to 72.3% [18] between 2018–2021, gaps remain in the uptake of required ANC visits, quality of care, uptake of delivery by skilled birth attendants, and other lifesaving MNCH services. The gaps between knowledge and use of ANC have been attributed to barriers such as financial, facility-related and personal reasons, and requirement for permission from the partner [18].

Group antenatal care (G-ANC) is a type of ANC based on the Centering Pregnancy model where health assessments, social support, and interactive learning are integrated and are at the core of the model [19]. Following new recommendations in 2016 by the WHO for a positive pregnancy experience [20], a randomized control trial (RCT) study conducted in Kenya and Nigeria showed that G-ANC was associated with higher facility-based delivery rates in Nigeria, higher proportion of women receiving quality ANC, and higher frequency of ANC visits [21].

Technical Advice Connect further corroborated these results by scaling up activities in Nigeria. More than 3, 000 health workers were trained in 1,542 health facilities across seven supported [7] supported states. A total of 1, 028, 766 pregnant women were enrolled in 81, 473 cohorts. Results show improved retention in care and increased uptake of key outcomes such as skilled birth attendance and post-partum family planning [22].

Based on these findings, G-ANC was prioritized as essential for improved maternal and child health and well-being and demonstrated as an effective and feasible antenatal care delivery model in LMIC [23]. However, despite this potential to improve uptake of MNCH services in LMICs, the intervention has been scarcely implemented in Nigeria.

The Centre for Integrated Health Programs (CIHP), between 2020–2021, with funding from the Bill & Melinda Gates Foundation (BMGF) through Technical Advice Connect (TAConnect), supported Kaduna State Government to adopt, adapt and sustain G-ANC service delivery model in selected PHFs. The goal of the project was to provide technical assistance to the Kaduna State Ministry of Health (SMOH), its State Primary Health Care Board (SPHCB) and Local Government Health Authorities to adopt, adapt and sustain G-ANC as an alternative model of ANC service delivery. The related objectives are summarized in the text box below. This paper aims to describe the implementation of G-ANC in Kaduna, the outputs, and lessons learned.

**Box 1.1. Group antenatal care project objectives**

- To increase the proportion of enrolled pregnant women achieving a minimum of eight ANC contacts.
- To increase uptake of IPTp using Sulfadoxine-Pyrimethamine (IPTp-SP) to at least four doses among pregnant women
- To increase the proportion of deliveries supervised by skilled birth attendants by at least 20%

### The concept of group antenatal care

G-ANC offers an alternative model to traditional ANC by participatory and facilitated learning, peer clinical assessment, sense of empowerment, and peer support consisting of three components; clinical health assessment, interactive learning, and community building [24]. G-ANC involves the formation of a cohort of 8–15 pregnant women having the same gestational age. The cohort engages in participatory facilitated learning supervised by a health care worker during G-ANC sessions using appropriate and tailored materials, with the pregnant women leading discussions and related activities. The grouping was done based on gestational age, the maternal age was not considered for cohort formation. As part of the clinical health assessment, the pregnant women conducted blood pressure (BP) measurement and weight examinations for their peers, which were documented in the client assessment card. The digital BP apparatus had a colour code which made readings easily interpretable, irrespective of their literacy level.

## Methodology

### Project location:

Kaduna State is in the Northwest geopolitical zone of Nigeria with 76% of the population living in rural areas concentrated in 19 Local Government Areas (LGA) [25], Giwa, Soba, Lere, Jema’a and Zango Kataf being the most densely populated rural LGA. Twenty-four percent of the population live in urban settings mainly in four LGA (Kaduna North, Kaduna South, Zaria and Sabon Gari) [25]. The state covers an area of 46,053 km2 and is bordered by Kano, Katsina, Zamfara, FCT, Nasarawa, and Plateau States [26].

**Facility Selection:** Primary Health Facilities (PHFs) were selected based on predetermined criteria including availability of at least four health care workers, 24 hours operation, capacity to conduct antenatal and delivery services, and availability of space within the facility to conduct group activities. A total of 255 PHFs were proposed and accepted for project implementation by the Kaduna SMOH and SPHCDB.

## Program description

The technical assistance (TA) provided to Kaduna State government was built on three key strategic approaches: 1) Facilitated skills transfer to the officials of SMOH and SPHCB on program management; 2) Clinical systems optimization of health facilities through technical assistance on outcome-focused quality mentoring; and 3) Sustainable and iterative systems appraisal using continuous quality improvement approaches. These strategies, and a costed implementation plan derived from these strategies, were reviewed, adopted and aligned with the Kaduna state Annual Operational Plan (AOP) for efficiency and sustainability

Our methodology was based on the implementation approach of the pragmatic cluster-randomized controlled trial in Kenya and Nigeria mentioned above with adaptations to existing health systems for sustainability. The approach involved a client-centered model [27] using the G-ANC platform to increase uptake of improved ANC and postnatal care (PNC) services including Malaria in Pregnancy (MiP) interventions (IPTp SP, increased use of ITN and early detection and treatment). Due to the COVID-19 pandemic, the implementation timeline, originally planned for 18 months, was extended by eight months. The supportive supervision plan was integrated into existing facility’s supervisory support visits for improved technical support by identified and trained Local Government-based clinical mentors. The key activities implemented for the project included stakeholders’ engagement, capacity building on G-ANC for Maternal Neonatal and Child Health (MNCH) HCW at the PHC, and demand creation using volunteer community mobilizers (VCMs) for referral in the community for G-ANC services.

**Advocacy visit and Stakeholders’ Engagement:** Multi-level advocacy visits to the leadership of the State Ministry of Health (SMOH) and State Primary Health Care Development Board (SPHCDB) were conducted for program support and adoptions for sustainable implementation. This led to the inclusion of the G-ANC activities into the State Annual Plan and adoption by the State Executive Council. Sensitization meetings were also held with health care providers, traditional leaders, and various stakeholders from the Local Government Area (LGA) and communities.

**Adaptation of Group ANC Materials:** For facilitating G-ANC sessions, we adopted four tools previously used by JHPIEGO in the implementation of G-ANC, namely; Facilitators Guide, Picture Cards, New Mothers Booklet, and Self-Assessment Card. These tools were reviewed and adapted to ensure cultural appropriateness and simplicity of content. The facilitator’s guide was adapted from the document by Lindsay Grenier et al [28].

**Capacity building and continuous supervision:** Effective implementation required that health care providers’ capacity to be built using a flexible training approach and continued supportive supervision. We used the *Low Dose High Frequency (LDHF)* [29,30] approach to rapidly train 735 health care providers comprising Nurses, Midwives and Community Health Extension Workers (CHEW) on G-ANC implementation across the selected 255 PHF. (Table 1). The training modules were delivered in three doses and spaced for one-month interval. This was to allow health care workers to absorb and apply the knowledge and skills acquired before the next module. Health workers were paid stipends for meals and incidentals during the training. However, no incentives were provided for the delivery of G-ANC services.

**Table 1:** Low dose high frequency training schedule used for step-down training.

DOSE	MEETING	FOCUS
**1**^**ST**^ **DOSE**	**Meeting 1**	Preventing Problems in pregnancy
		Malaria in pregnancy
		Road to life/Road to death
	**Meeting 2**	Danger signs and problems in pregnancy
		Introduction to birth preparedness
	**Meeting 3**	Birth Preparedness and Complication Readiness (BPCR)
		Post-natal care (PNC) and healthy timing & spacing of pregnancy
**2**^**ND**^ **DOSE**	**Meeting 4**	Birth spacing methods
		Respectful Maternity Care
		Exclusive Breastfeeding
	**Meeting 5**	Gender-based violence/Intimate Partner Violence
		What to expect in labor
		Support in labor and recognizing baby problems
**3**^**RD**^ **DOSE**	**Meeting 6**	Malaria in Pregnancy (MiP)
		Recognizing women problems after birth
	**Meeting 7**	Hope for labor & Malaria in Pregnancy (MiP)

Additionally, a 5-day training on Clinical System Mentoring (CSM)) – a health system diagnostic, support and staff mentoring skill acquisition capacity building model – was conducted for 33 LGA MNCH coordinators and directors from SMOH, SPCDB, and Planning, Budget and Coordination (PBC) units. This training equipped LGA clinical mentors and the state leadership team to provide technical support in the implementation of G-ANC and integrated services**.** Five hundred and eighty-one VCMs were also trained to improve MNCH service uptake expanding the scope of existing VCMs on MNCH activities. Each of VCMs visited 30 households within communities on a bi-weekly basis to sensitize community members.

### Group antenatal care session implementation

For the G-ANC session, three core materials were used during every G-ANC visit: the facilitator’s guide, the picture cards and the mother’s take home booklet. The last two were used by both facilitators and the clients. Each session followed the same overall framework. This helped both facilitators and group members know what to expect for better learning. At the first group meeting, the facilitator taught pregnant women on how to use the blood pressure apparatus, weighing scale, illustration care card, self-assessment cards and documentation of findings. Pregnant women were taught to recognize danger signs and symptoms in pregnancy, improve personal hygiene and mobilization skills to help get everyone together to engage in activities. The group meetings were held monthly to discuss maternal and child health issues (Table 2). Pregnant women who were 32 weeks or more met biweekly until delivery.

**Table 2:** Group ANC visit schedule.

CONTACT	SESSION	TIMING	THEME/SESSION
1	**First Contact**	**Booking and Grouping**	
2	Session 1	12–20 weeks	Introduction to group care
			Preventing problems in pregnancy
3	Session 2	20–24 weeks	Recognizing problems in pregnancy (Discuss Nutrition)
			Joint planning on topics for male engagement in Meeting 3
4	Session 3	24–28 weeks	Role of partner in a positive pregnancy experience
			Birth preparedness and complication readiness (BP/CR)
			Benefits of hospital delivery (cultural misconceptions to hospital delivery)
			Importance and Timing of Post-natal care
			Healthy timing and spacing of pregnancies (HTSP)
5	Session 4	28–32 weeks	Labor signs
			Birth Spacing
			Exclusive breast feeding
6	Session 5	32–34 weeks	Labor and birth: what to expect; visit labor ward
			Preventing problems after birth (Discuss Immunization of mother and child)
7	Session 6	34–36 weeks	Recognizing baby problems
			Support in labor
8	Session 7	36–40 weeks	Recognizing postpartum maternal problems
			Final birth planning

**Monitoring and evaluation:** CIHP strategic information staff in collaboration with the SPHCDB monitoring and evaluation team adapted existing documentation tools on ANC, HIV testing Services, family planning other MNCH tools for the implementation of G-ANC. The tools were used by trained G-ANC staff to document the group sessions. CIHP supported the State to train Health Management Information System (HMIS) Officers on the National Health Management Information System tools (2019 version) and G-ANC documentation. The project also built the capacities of Monitoring and Evaluation officers on District Health Information Software (DHIS2) and data analysis. Program data was entered into the DHIS2 by the HMIS Officers every month, aggregated at the facility level. Aggregated data retrieved monthly from DHIS2 was analyzed to monitor program indicators’ trends for decision making and addressing identified gaps. An end-line mixed-methods evaluation was conducted at the end of implementation to assess the extent of achievements of the project objectives.

### The Reproductive, Maternal, Newborn, and Child Health+ Nutrition (RMNCH+N) dashboard analytics portal

As part of the technical and programmatic support for the G-ANC grant and to increase access to high-quality health data in Kaduna State for Reproductive, Maternal, Neonatal and Child Health + Nutrition (RMNCHN+N), CIHP supported the state to develop and deploy a thematic focused dashboard on tracking RMNCHN+N interventions across the continuum of care. One of the core objectives of this dashboard is to monitor and evaluate health outcomes and improve the use of reproductive, maternal, newborn and child health routine service data to inform decisions at the facility, LGA, and state levels. The dashboard analytics was developed on Tableau software application version 2021.4 [31] embedded in a web portal that is accessible from any web browser device enabled. The dashboard visualizes, shares and displays data on Reproductive, Maternal, Newborn, and Child Health (RMNCH). This covers the health concerns and interventions across the life course involving women before and during pregnancy; newborns (the first 28 days of life), and children to their fifth birthday. This dashboard identifies and examines over 250 indicators across Kaduna State cutting across data from State, LGAs, Wards down to facility levels for which data are available from the period 2017 – 2021. The dashboard contains disaggregation such as age, gender, and other variables using a filter that can be easily navigated and displayed on the visuals/charts.

### Data analysis

Quantitative program data (e.g., ANC, IPT, and post-partum contraceptive uptake) were downloaded from the DHIS, cleaned and analyzed using Excel. Frequencies and percentages were presented using frequency distribution tables. Percentage changes in specific project indicators comparing baseline and end line were calculated and also presented. Furthermore, we conducted desk review of the program report, using a data abstraction template, to analyze process indicators (e.g., number and type of trainings conducted, number of persons trained by category of participants and type of training)

The limitation of the intervention was the grouping of pregnant women based on maternal age, which would have helped in the disaggregation of adult mothers and young mothers (<19 and 20–24 years).

### Ethical considerations

Individual ANC was already established across facilities at the commencement of the G-ANC project. Pregnant women were introduced to group antenatal session and recruitment was optional with the opportunity to opt out at any time. All ANC services were offered to both individual and group ANC participants at their own choice without any coercion or incentives to enroll in the G-ANC platform. All clients who enrolled in the G-ANC were provided informed verbal consent. Data used for analysis were collected from the ANC facility registers in aggregated format and did not include individual level data or personal identifiable information, thereby preserving the confidentiality of clients’ information.

## Key program results

The project was implemented in 255 primary health facilities in Kaduna State.. A total of 765 HCW were trained who facilitated 26,769 G-ANC sessions between January 2020-Februrary 2022 for 309,751 pregnant women who formed a total for 23,220 cohorts. A total of 78,015 [25] of pregnant women were enrolled for ANC by VCMs over this period (Table 3).

**Table 3:** Group antenatal care data summary for project indicators – January 2020-Februray 2022 (Data source – program reports).

Output (Process) Indicators	Program Target	Total
Number of HFs conducting G-ANC Model	255	255
Number of HCWs trained as G-ANC facilitators in G-ANC supported facilities	**765**	**765**
Number of HCWs trained on CQI in G-ANC supported facilities	261	261
Number of Persons trained as G-ANC Clinical Mentors in Kaduna State	92	92
Number of Persons trained as G-ANC Master Trainers in Kaduna State	20	20
Number of Persons trained as CQI Master Trainers in Kaduna State	33	33
Number of Persons trained on Clinical Systems Mentoring as Master Trainers in Kaduna State	33	33
Number of Persons Trained on the National Health Management Information System (NHMIS)	33	33
Number of Persons Trained on Data Management (M&E Training)	26	26
Number of trained volunteer community mobilizers that referred at least a PW to HF during reporting period. (Monthly Average)	512	512
Number of PW that enrolled for ANC referred by volunteer community mobilizers in the reporting period	78015	78015
Total number of cohorts of pregnant women formed till March 2022		23220
Total number of pregnant women enrolled in G-ANC till March 2022		309751
Total Deliveries in the facility		101789

All (100%) of health workers who worked in implementing facilities were trained as G-ANC facilitators.

Post-intervention, comparing baseline to end-line there was more than two-fold increase (228%) in uptake of four-doses of Intermittent Preventive Treatment (IPT) of malaria (6.5% vs 21.5%), 152% increase in acceptance of post-partum contraceptives (11.4% vs 28.9%), and 66.4% increase in 8^th^ ANC visit (10.2% vs 16.9%). (Table 4).

**Table 4:** Results of key program service delivery indicators comparing 12 months pre- and post-G-ANC intervention periods (Data Source – District Health Information Software).

Indicators	Baseline (Jan 2020 - Dec 2020)		Implementation (Jan 2021 - Dec 2021)		% Change from baseline*
	n	%	n	%	%
**Number of pregnant women enrolled for antenatal care (Had ANC 1st visit)**	N = 179033		N = 213457		
**Number of pregnant women attending ANC 1st Visit at gestational age less than 20 weeks**	55141	30.8	63625	29.8	-3.2
**Number of pregnant women with ANC 4th visit**	99782	55.7	87292	40.9	-26.6
**Number of pregnant women with ANC 8th visit**	18228	10.2	36161	16.9	66.4
**Number of pregnant women receiving 4 doses of intermittent preventive treatment (SP)**	11716	6.5	45848	21.5	228.2
**Number of deliveries in facility**	N = 73563		N = 81991		
**Number of pregnant women delivered in facility who accepted postpartum family planning before discharge**	8419	11.4	23662	28.9	152.2

*Note: % changes from baseline were calculated by first obtaining the difference in baseline and endline percentage, then dividing the difference by baseline percentage, and finally multiplying by 100.

Finally, the Kaduna State Government (KSG) updated the ANC policy and annual operational plans to incorporate G-ANC activities to be guided by a sustainability plan. The KSG further advocated for countrywide adoption of G-ANC at the 2021 National Council for Health meeting. Data as shown in the result displayed an increase in all the quality indicators measured.

## Discussions

The study described the implementation of G-ANC in 255 PHF in Kaduna State based on adapted practices previously tested in a randomized cluster trial (RCT) conducted in Nasarawa State, Nigeria, and Kisumu and Machakos Counties in Kenya.[21] Although this project adopted the original G-ANC RCT protocol, local nuances and client’s preferences resulted in the variation in the G-ANC gestational age of recruitment (all pregnant women irrespective of gestational age versus 20–24 weeks in the RCT) into G-ANC. Another variation was the addition of a community component of malaria prevention in pregnancy with the use of VCMs which was not included in the RCT.

The total expected number of pregnant women during the intervention period in the state was estimated at 249,936. The intervention reported an enrollment of 309,751 pregnant women with a coverage rate well over 100%, suggesting that the intervention covered a significant scope at the state level. Several factors may explain this over-coverage, including the possibility that pregnant women from neighboring states accessed services at the intervention sites. Additionally, the estimated number of pregnant women may have underrepresented the actual number of pregnant women, possibly due to population growth or limitation in available demographic data due to outdated national census population figures often for population projections.

Beyond the numerical coverage, the intervention demonstrated improvements in service uptake and maternal health outcomes. Specifically, there was improvement in antenatal visits, skilled birth deliveries, and uptake of IPTp pregnancy and family planning services, that are key to the attainment of better maternal health outcomes. Although there was an increase in the absolute number of women attending the 4th ANC visit, the proportion relative to total ANC clients decreased. This paradox can be explained by the significant rise in ANC enrollment following the G-ANC model rollout. Many women initiated ANC later in pregnancy, thus missing earlier visits like the 4th but remaining engaged long enough to complete later contacts such as the 8th. The observed increase in 8th visit attendance reflects improved client retention facilitated by the structured, peer-supported nature of G-ANC. Meanwhile, the proportional drop at the 4th visit likely results from late booking, visit scheduling patterns, and differences in how contacts were recorded. This underscores the need to not only increase early ANC initiation but also to improve continuity and tracking of care across the full ANC schedule.

The results of program outputs showed that ANC utilization was lower compared with national averages reported in 2018 NDHS [32]. The 41% achieved post-intervention was substantially lower than the national average of 58% of ANC 4^th^ visit. Furthermore, a notable increase was observed a year post-implementation for 8^th^ ANC visit, although this 17% increase post-intervention was less than the national average of 21% in 2018. Similarly, although IPTp coverage gains were modest after the implementation of G-ANC, they reflect measurable progress toward national goals.

For indicators related to the quality of service received by the pregnant women, the rate of completion of four doses of SP and acceptance of post-partum family planning among women who had facility delivery increased post-intervention. This observation suggests the potential for the G-ANC to improve these key qualitative components of maternal care, however the relatively lower utilization compared to national averages suggest the need to further identify barriers to maximizing the effect of G-ANC.

The project was implemented on the platforms of strong partnership, synergies and collaboration with government, Reproductive, Maternal, and Child Health task team members, community gate keepers, and opinion leaders. These platforms were critical to ensuring sustainability and entrenchment into the health system as a comprehensive strategy for improving and sustaining RMNCH outcomes [33]. To sustain the implementation, all costed G-ANC activities were included in the health component of Kaduna State’s Annual Operational Plan and budgetary allocation for procurement of G-ANC equipment and supplies. The adoption of the G-ANC by Kaduna State as a service delivery model for MNCH prompted the State to propose its adoption to the Nigerian National Health Council for MNCH services.

The implementation timeline was originally planned for 18 months (Jan 2020 to June 2021) but was extended to February 2022 due to COVID 19 pandemic. Implementation was at the height of the COVID 19 pandemic and even before vaccines were created, so infection control measures were instituted at meetings and training while virtual meetings were used to augment physical meetings. During the pandemic, there was decline in hospital attendance in Kaduna State, owing to the stricter enforcement of a lockdown compare to other states in Nigeria where there was substantial noncompliance to the lockdown restrictions [34]. However, overall, the G-ANC recorded no significant reduction in ANC services utilization due to a rebound hospital attendance especially in hospital deliveries after easing of the lock down [35]. Cultural context such as family bounding and desire to socialize after the lock down might have played a role in the rebound.

Personalized group care that the G-ANC modeled, created a sense of camaraderie and a feeling of empowerment for the pregnant women. Take home picture cards and messages were simplified, relatable with actionable information, group learning was motivation for adapting the right health behavior with improved self-assessment and accountability. Engagement during waiting time reduced the illusion of prolonged stay in the hospital and appeared to increase the pregnant women’s positive experience. A substantial part of the patient interaction was devolved to the group thus reducing the workload on the health care provider and the associated burnout. Transparent and respectable community engagement coupled with the recruitment of VCMs, made up of respectable community members, improved community acceptance and trust, leading to meaningful contribution in addressing contextual barrier.

During CSM training the motivation to use data for decision and garner consensus was inculcated on the clinical mentors and facilities leads as well as the capacity to defining problems through data and apply little test of change to address identified problems. The NHMIS was strengthened through capacity building and the provision of new data capturing tools leading to improved reporting, while the data dashboard made analyzed data available for decision makers.

In light of the ever-present threat of minimal government financial commitment and delayed fund release due to prioritization of needs, enrollment and linkage to the state health insurance scheme and other local funding sources can support sustainability and ownership. Sustainability and funding must be brought to the forefront given that maternal and child health is an issue of social security and overall community health. The better framing of these issues will help set the agenda in government engagement and focus directions in planning and measurement of impact. Infrastructure is another challenge to be addressed before scale up so that state-wide impact can be felt. This is crucial as only a fraction of the over 1600 PHC and private facilities in the state were involved in G-ANC as the derelict state of many facilities prevented their inclusion.

To sustain this model of care, early and transparent communication/advocacy to relevant state government agencies, especially the state Planning and Budget commission, is necessary. This will enable co-creation of key performance indicators on transition road map; linkage to sustainable domestic funding, agreement of role and responsibilities of stakeholders, availability of commodity supplies, and essential facility repairs. Discussing roles, responsibilities and collateral benefits of Volunteer community volunteers like the VCMs in the structure community health interventions of wider maternal and child health outcomes is key to sustaining community intervention [36]. Where established community structures like the Community Health Influencers, Promoters and Services (CHIPS) [37] and ward development committees [38,39] exist they should be strengthened to provide a more structured platform for community intervention.

Opportunities exist in community G-ANC implementation, as this will widen the scope, reach and improve ANC services the participation of community structures. It will also provide contextual intervention and experience of the age-old problem of hospital visits by pregnant women. It will likewise strengthen community structures and cultural institutions in the ownership and shaping of health care demand and access by improving acceptability.

Despite the promising outcomes, the scale-up of G-ANC across Kaduna State revealed persistent challenges that merit deeper consideration. These include limited infrastructure in many PHCs, human resource constraints, and logistical barriers such as inconsistent commodity supply and weak referral systems. Furthermore, entrenched socio-cultural norms, late ANC booking, and lack of male partner involvement continue to limit early engagement and continuity of care. Policy implications of these findings suggest the need for stronger integration of G-ANC into national and sub-national maternal health strategies, with dedicated funding, harmonized training for frontline workers, and alignment with community-based programs like the CHIPS.

## Limitations

The project was implemented in the 255 PHC that met the predetermined selection criteria out of more than 1,600 PHC in the state. The basis of this selection may make them different from other PHCs so that measured outputs might have been different for these other facilities where G-ANC was not implemented. In addition, the analysis in this paper did not account for the effect of seasonality or for the clustering effect of pregnant women attending the same health facilities. Finally, the paper did not report the outcomes or expected impact of the project. This is to be published in another paper which will also provide useful insights into the barriers and facilitators of G-ANC implementation through the perceptions of beneficiaries, implementers, and government health officials which can inform better implementation in scaling up to other PHCs.

## Recommendations

Group ANC should be introduced to all health facilities to improve antenatal visits, skilled birth deliveries, and uptake of IPTp pregnancy and family planning services. In preparation for this scaleup, it is essential that health facilities are equipped with necessary infrastructure and equipment, including adequate space availability, for effective G-ANC sessions in the health facilities. Intersectoral collaboration to empower and educate communities on early registration of pregnancy for prompt detection of complications will be key to improve uptake of services. Additionally, the government through the Ministry of Health should make adequate funds available to procure clinical items for self-assessment. Further studies should explore the implementation challenges, enablers and promoters of G-ANC, especially in more diverse settings. In addition, such studies should measure the impact of G-ANC on reducing maternal and neonatal mortality in the state after a few years of adoption which can be used to provide evidence for the cost-effectiveness of the intervention.
